# Clinical Outcomes between P1 and P0 Lesions for Obscure Gastrointestinal Bleeding with Negative Computed Tomography and Capsule Endoscopy

**DOI:** 10.3390/diagnostics11040657

**Published:** 2021-04-06

**Authors:** Young Kyu Cho, Heesu Park, Jung Rock Moon, Seong Ran Jeon, Hyun Gun Kim, Tae Hee Lee, Junseok Park, Jin-Oh Kim, Joon Seong Lee, Hyeon Jeong Goong, Bong Min Ko, Suyeon Park

**Affiliations:** 1 Digestive Disease Center, Institute for Digestive Research, Soonchunhyang University College of Medicine, 59 Daesagwan-ro, Yongsan-Gu, Seoul 04401, Korea; 109133@schmc.ac.kr (Y.K.C.); phs759@hanmail.net (H.P.); ukino816@gmail.com (J.R.M.); medgun@schmc.ac.kr (H.G.K.); iman0825@naver.com (T.H.L.); junspark@schmc.ac.kr (J.P.); jokim@schmc.ac.kr (J.-O.K.); joonlee@schmc.ac.kr (J.S.L.); goong@schmc.ac.kr (H.J.G.); kopa9445@schmc.ac.kr (B.M.K.); 2 Department of biostatistics, Soonchunhyang University College of Medicine, 59 Daesagwan-ro, Yongsan-Gu, Seoul 04401, Korea; suyeon1002@schmc.ac.kr

**Keywords:** obscure gastrointestinal bleeding, computed tomography, capsule endoscopy, rebleeding

## Abstract

Background: A simple classification for the relevance of lesions (P0, P1, and P2; no bleeding potential, less likely to bleed, and more likely to bleed, respectively) based on capsule endoscopy (CE) findings has been used. This study aimed at investigating rebleeding rates and predictive factors of P0 and P1 lesions after obtaining negative findings in both, CE and computed tomography (CT), for patients with obscure gastrointestinal bleeding (OGIB). Methods: Among 193 patients resulted in negative CE findings defined as P0 or P1 lesions, 84 patients with negative results on CT images were enrolled in this study. The rebleeding rates and predictive factors were assessed in the P0 and P1 groups. Results: Overall rebleeding rate in patients with negative CT and CE was 17.9%; 18.4% in the P0 group; 17.4% in the P1 group within a median follow-up duration of 18.5 months. In the P0 and P1 groups, the cumulative rebleeding rates were 9.2%, 25.4%, and 25.4%, and 6.9%, 11.8%, and 18.6% at 12, 24, and 60 months, respectively (*p* = 0.97). There were no independent rebleeding associated factors in the P0 group, whereas Charlson comorbidity index (CCI) (hazard ratio (HR) = 2.019, 95% confidence interval (CI): 1.158–3.519, *p* = 0.013), and initial low hemoglobin (Hb) level (<8 g/dL) (HR = 15.085, 95% CI: 1.182–192.514, *p* = 0.037) were independent predictive factors responsible for rebleeding in the P1 group. Conclusions: Despite having negative findings on CT and CE, patients with OGIB have a significant potential rebleeding risk. Although there was no significant difference in rebleeding rates between the P0 and P1 groups on CE, the P1 group, with CCI or low initial Hb level, should be cautiously observed after the first bleeding episode.

## 1. Introduction

Obscure gastrointestinal bleeding (OGIB) represents approximately 5–10% of all gastrointestinal bleedings [1,2]. In most guidelines of OGIB, capsule endoscopy (CE) is considered as the first-line modality [1,3,4,5]. The P0-P2 classification (no bleeding potential, less likely to bleed, and more likely to bleed, respectively) based on CE findings has been used. According to Korean guidelines, contrast-enhanced abdominal computed tomography (CT) as well as CE are equally considered as first-line modalities [4]. Therefore, when CE is contraindicated, unavailable, or resulting in a negative finding, abdominal CT can be considered as an alternative for the initial examination of OGIB [4,6,7]. However, Japanese guidelines recommend contrast-enhanced CT as the first-line diagnostic modality for OGIB since initiation [8]. CT first algorithm may be useful owing to the ease of use, and ability to obtain rapid results or luminal/extraluminal findings that cannot be detected on CE. In cases of absence of abnormal findings on the abdominal CT, subsequent CE is recommended [8].

In patients with OGIB, when CE results are negative, lower rebleeding rates of 4.8–35.7% have been reported compared to 6.7–46.6% for those with positive CE results [9,10,11,12,13,14,15]. A recent meta-analysis reported that the pooled rebleeding rate after negative CE result was significantly lower compared to that after positive CE result [16]. Therefore, patients with OGIB after negative CE can be conservatively treated based on these results [5]. However, several studies reported that rebleeding rate after negative CE in patients with OGIB was not low [9,13,17]. Thus, rebleeding rate in patients with OGIB with negative CE result is still contradictory.

In addition, there are few data on the clinical outcomes of patients with negative CE (defined as P0 and P1) and abdominal CT findings. This study aimed at investigating rebleeding rates and predictive factors of P0 and P1 lesions after negative findings in both, CT and CE, for patients with OGIB.

## 2. Methods

### 2.1. Study Design and Patients

**Figure 1:**
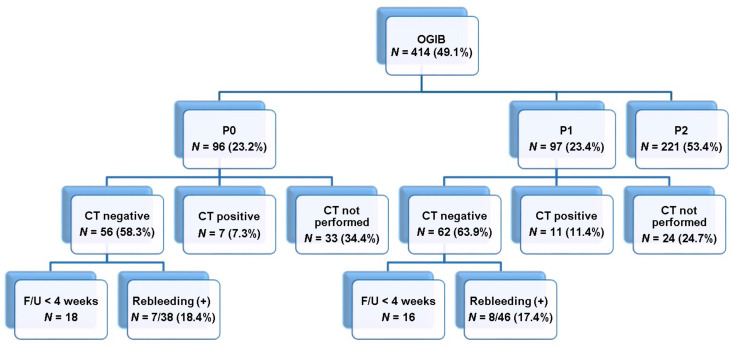
Enrollment flowchart.

We retrospectively analyzed the clinical data of 844 patients who underwent consecutive CE (PillCam^TM^; Covidien–Medtronic, Dublin, Ireland or MiroCam^TM^; IntroMedic Co., Seoul, Korea) conducted prospectively from March 2003 to August 2019 at the Soonchunhyang University Hospital. Among them, 414 CE studies were performed for OGIB investigation, with 193 resulting in negative CE findings. Overall, 84 patients with negative results on abdominal CT and subsequent follow-up period of a minimum of 4 weeks, were enrolled in this study (Figure 1). The primary endpoint was the rebleeding rate according to the P0 and P1 lesions on CE in patients with OGIB and negative findings in both, CT and CE. The secondary endpoint was to identify the risk factors associated with rebleeding of P0 and P1 lesions. The study was conducted in accordance with the Declaration of Helsinki. The protocol was approved by our institutional review board (IRB), and patient consent to participate was waived in accordance with the IRB (SCHUH 2020-06-021).

Medications (antiplatelet (anti-PLT) agent, anticoagulant, and nonsteroidal anti-inflammatory drugs NSAIDs)) associated with gastrointestinal bleeding (GIB) were temporarily discontinued at the initial GIB episode. The reuse of these medications was defined as persistent anti-PLT agent, anticoagulant, or NSAIDs use. Overt OGIB was defined as bleeding that persisted or recurred after initial negative upper and lower endoscopies. Occult OGIB was defined as either iron-deficiency anemia (IDA), positive fecal immunochemical test, or combination of both [18]. Rebleeding was defined as evidence of GIB (overt bleeding or a fall in hemoglobin (Hb) level ≥2 g/dL compared with the baseline value and without other causes of decline in Hb) without bleeding focus in the repeat upper and lower endoscopies for a minimum of 4 weeks after the first bleeding [9,19].

### 2.2. Capsule Endoscopy and Abdominal CT Procedures

PillCam^TM^ (Covidien–Medtronic, Dublin, Ireland) or MiroCam^TM^ (IntroMedic Co., Seoul, Korea) were used for the procedures. Polyethylene glycol (PEG)-based bowel preparation including a minimum fasting of 8 h was performed before CE. For identification of gastric retention, simple abdomen radiography was performed 2 h after CE. The quality of bowel preparation was categorized as follows: excellent, good, fair, or poor [20]. Regarding the image quality of CE, excellent and good preparations were considered adequate, whereas fair and poor preparations were considered inadequate [21]. Two board-certified expert endoscopists (JSR and KHG) reviewed all CE images. Disagreement on any dubious image was solved by joint discussion. We classified the findings from P0 to P2. P0 (visible submucosal vein, diverticula without bleeding) and P1 (red spots, small isolated angioectasia or erosion, and edematous vessel) lesions were considered as negative findings, whereas P2 lesions (typical angioectasia, large ulceration, tumor, or varix) were considered as positive with bleeding focus [22].

Conventional contrast enhanced abdominal CT and CT enterography (CTE) were included in the abdominal CT. Before CTE, 1800 mL of 4.4% (weight/volume) sorbitol solution was administered for 1 h.

### 2.3. Statistical Analysis

All statistical analyses were applied using IBM SPSS Statistics version 21.0 (SPSS Inc., Chicago, IL, USA). All continuous and categorical variables were compared using a two-tailed Student’s *t*-test and the chi-squared test, respectively. The cumulative rebleeding rates during follow-up were calculated using the Kaplan–Meier method. Predictive factors associated with rebleeding were assessed through univariate and multivariate Cox logistic regression analysis. Charlson comorbidity index (CCI) was analyzed as a continuous variable using Cox logistic regression analysis. In the univariate analysis, variables with a *p*-value of <0.1 were included in the multivariate analysis. A *p*-value of <0.05 was considered statistically significant.

## 3. Results

### 3.1. Baseline Characteristics

The mean age was 57.9 years and 58.3% (49/84) of all study participants were male. The first bleeding was overt type in the majority of patients (67/84, 79.8%). Drugs that increase bleeding risk were treated in 27.4% (23/84) of all patients. The lowest Hb level at the first episode (mean ± SD) was 8.6 ± 2.6 g/dL and the unit of transfusion (pack red blood cells (pRBC), mean ± SD) was 1.1 ± 1.9. Mean follow up duration was 35.9 months. The patient’s demographic data with negative CT and CE are summarized in Table 1.

### 3.2. Capsule Endoscopy-Related Data

CE-related data are summarized in Table 2. Complete examination of CE was achieved in 85.7% (72/84) patients. No CE retention was observed in all patients. P0 and P1 findings of CE were obtained in 54.8% (46/84) and 45.2% (38/84), respectively. Considering the P1 findings, red spot(s) or small isolated angioectasia were the most common (22/46, 47.8%), followed by small isolated erosion (18/46, 39.1%), and combined lesions (1/46, 13.0%). There were no statistically significant differences except aspirin use at the first bleeding (2.6 vs. 19.6%, *p* = 0.020) between P0 and P1 groups (Table 3).

### 3.3. Rebleeding Analysis

**Figure 2:**
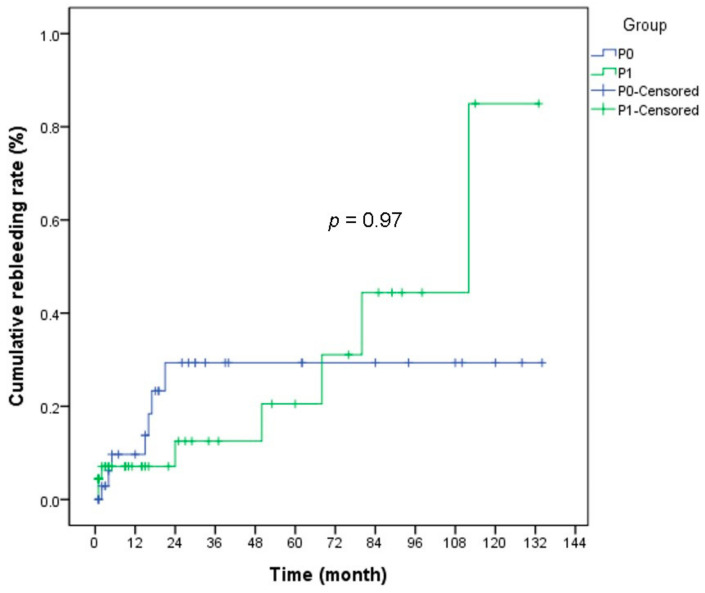
The cumulative rebleeding rates according to P0 and P1 lesions on capsule endoscopy. In the P0 group, the cumulative rebleeding rates at 12, 24, and 60 months were 9.2% 25.4%, and 25.4%, respectively. The cumulative rebleeding rates of the P1 group at 12, 24, and 60 months were 6.9%, 11.8%, and 18.6%, respectively. There was no significant difference between the P0 and P1 groups (*p* = 0.97).

In 84 patients with negative findings for both CT and CE, overall rebleeding rates were 17.9% (15/84), 18.4% in P0 group (7/38), and17.4% in P1 group (8/46) within a median follow up duration of 18.5 (range 1–134) months. In P0 and P1 groups, the mean time interval between the first bleeding episode and rebleeding were 11.4 months and 42.3 months, respectively (Table 3). The cumulative rebleeding rates at 12, 24, and 60 months in follow up were 8.0%, 18.9%, and 22.6%, respectively. In the P0 group, the cumulative rebleeding rates were 9.2% and 25.4% at 12 and 24 months, respectively. The cumulative rebleeding rate of the P1 group was 6.9%, 11.8%, and 18.6% at 12, 24, and 60 months, respectively. There was no significant difference between the two groups (*p* = 0.97) (Figure 2).

### 3.4. Rebleeding Risk Factors Analysis

In the univariate analysis of the P0 groups, older age >65 years, aspirin use, initial Hb level <8 g/dL, and reuse of bleeding-related drugs were associated with rebleeding. However, there were no rebleeding-associated factors in the multivariate analysis. In the univariate analysis of the P1 group, CCI, liver cirrhosis (LC), initial Hb level <8 g/dL, and pRBC transfusion >2 pints were associated with rebleeding. Both CCI (hazard ratio (HR) = 2.019, 95% confidence interval (CI): 1.158–3.519, *p* = 0.013) and initial low Hb level (<8 g/dL) (HR = 15.085, 95% CI: 1.182–192.514, *p* = 0.037) were independent predictive factors responsible for rebleeding (Table 4). Multivariate analysis also revealed that both CCI (HR = 1.366, 95% CI: 1.016–1.836, *p* = 0.039) and initial low Hb level (HR = 9.001, 95% CI: 1.875–43.202, *p* = 0.006) were independent predictive factors responsible for rebleeding in patients with OGIB, negative for both CT and CE. Comparison of characteristics between the non-rebleeding and rebleeding groups are summarized in Supplemetary Table S1. Cox regression analyses of factors associated with rebleeding in patients with negative CT and CE are listed in Supplementary Table S2.

### 3.5. Clinical Outcome and Treatment of Rebleeding Patients

Of 84 patients with negative findings for both CT and CE, 15 patients experienced rebleeding. No bleeding focus was found in any patient on conventional upper and lower endoscopy. Among them, 11 patients underwent additional small bowel (SB) evaluation (CE, double-balloon enteroscopy (DBE), push enteroscopy, CTE, or angiography) for recurrent OGIB. Although repeated CE examination was performed, the focus of rebleeding was not found in 18.1% (2/11) patients, and they recovered spontaneously after receiving conservative treatment. The remaining nine patients were diagnosed as NSAIDs-induced enteropathy (*n* = 2) and SB angioectasia (*n* = 2) by CE and DBE; ileal hemorrhagic erosion (*n* = 1) by CE; either ulcer of duodenal third portion, proximal jejunal, or both (*n* = 3) by push enteroscopy; and jejunal varices (*n* = 1) by angiography. The patient with jejunal varices underwent angiographic embolization, whereas the remaining eight patients were treated conservatively.

## 4. Discussion

This study assessed rebleeding rate and risk factors in patients with OGIB with negative findings in both, CT and CE. This study was initiated based on two hypotheses; a significantly potential rebleeding risk observed even in OGIB patients with negative CT and CE, and the clinical outcomes of the P1 group varied compared to the P0 group. To our knowledge, this is the first study to analyze clinical outcomes according to P0 and P1 lesions in patients with OGIB with negative CT and CE.

The current guidelines recommend CE to be considered as a first-line modality for SB evaluation in patients with OGIB. Contrast-enhanced CT has been considered as a complementary diagnostic aid to CE in patients with OGIB [1,4,23,24]. Our previous study reported that the diagnostic yield of patients who underwent CT before CE in OGIB was 16.0% [25]. According to Japanese guidelines, the CT first algorithm was recommended to exclude SB lesions found in CT before CE in patients with OGIB [8]. Reflecting these points, we included OGIB patients with negative findings in CT as well as CE to evaluate rebleeding rate and risk factors. In our study, overall rebleeding rate in patients with OGIB patients with negative CT and CE was 17.9% within a median follow-up duration of 18.5 months. Rebleeding rate for patients with OGIB with long term follow-up after negative CE has been reported up to 36% [9]. In a study including a large number of patients with negative CE (*n* = 207), rebleeding was observed in 16.4% of patients [11]. The discrepancies of rebleeding rates in previous studies can be explained by differences in the number of enrolled patients, selection of patients, short duration of follow up, or subsequent treatment. A recent meta-analysis reported that the overall pooled rebleeding rate after negative CE was 19% [16]. These results are in accordance with our results. False negative results of CE can occur due to the limited visual field of CE, poor bowel preparation, inadequate luminal distention, rapid passage around the proximal small bowel, and the lesions where bleeding has stopped spontaneously during CE [26,27]. It implies that negative findings in both, CT and CE, in patients with OGIB, do not guarantee lower rebleeding rates during long term follow-up.

We also assumed that the rebleeding rate of the P0 group that was true negative was lower than that of the P1 group. Contrary to our expectations, in this study, there was no significant difference of rebleeding rate (18.4% vs. 17.4%) between the P0 and P1 groups. In a study performed between P0 (*n* = 63) and P1 (*n* = 37) lesions on CE in patients with IDA, subgroup analysis of 87 patients with a longer follow-up duration (>1 year) showed that rebleeding or IDA recurrence was not significantly different between the P0 and P1 lesions (21.6% vs. 37.0%, *p* = 0.349) [28]. The result of our study enrolled patients with OGIB with negative CT and CE, which is similar to the previously published results [28]. In the above-mentioned study, the causative lesions for IDA were diagnosed using conventional endoscopies [28]. Whereas in our study, additional SB evaluation was conducted in 81.8% (9/11) of the patients for diagnosis of recurrent SB bleeding. This suggests that the causes of rebleeding in OGIB patients with negative CT and CE were not overlooked in conventional endoscopies, but in true SB lesions.

Although it showed only statistical tendency (11.4 vs. 42.3 months, *p* = 0.078), the mean time interval between the first bleeding episode and rebleeding of P1 lesions was longer than that of P0. The difference in the cumulative rebleeding rates between the P0 and P1 groups were not statistically significant. The reasons for this are unclear. However, the presence of P0 lesions on CE of patients with negative CT and CE may lead to less clinical follow-up or intensive medical treatments. The P0 findings were possibly false negative on CE. Although patients with OGIB with negative CT and CE revealed the presence of P0 lesions on CE, it did not indicate less clinical concern. In addition, in the P1 group, a longer period (>2 years) of careful observation to identify rebleeding could be necessary depending on the case.

Several factors such as older age, anticoagulants and anti-PLT agent, presence of comorbidities, overt OGIB, initial low Hb, higher transfusion requirement, or continued use of anticoagulants are considered as risk factors associated with rebleeding [10,13,14,15,16,29]. In our study, CCI (burden of comorbidities) (HR = 1.3669, 95% CI: 1.016–1.836, *p* = 0.039) and initial low Hb (HR = 9.001, 95% CI: 1.875–43.202, *p* = 0.006) were also associated independently with rebleeding in OGIB patients with negative CT and CE. Similar results were observed in P1 lesions, whereas these risk factors were not associated with rebleeding in the P0 lesions. NSAIDs, anticoagulants, and anti-PLT agents have been associated with SB bleeding. In our previous study, the reuse of NSAIDs after initial SB bleeding was an independent rebleeding risk factor in patients with NSAIDs-induced enteropathy [30]. Drugs that increase bleeding risk were treated in 27.4% (23/84) of patients in this study. Of the 23 patients, 14 (60.8%) resumed taking the bleeding-related drugs due to underlying diseases; however, drug resumption was not linked independently to rebleeding in the P0 and P1 groups. Drug types, as well as reuse of bleeding-related drugs, may be responsible of rebleeding. However, statistical analysis of drug type and reuse of bleeding-related drugs was not possible because of fewer patients taking bleeding-related drugs in this study than in other studies.

We recognize some limitations of our study. First, this study was a retrospective analysis at a university hospital with a small number of patients. Therefore, a large and prospective trial will be needed because our conclusions on clinical outcomes between P0 and P1 lesions in patients with OGIB may be limited. However, unlike other studies, we selected only patients confirmed with negative findings on CT and CE to analyze the difference of clinical outcomes of P0 and P1 lesions in patients with OGIB. Second, small isolated angioectasia as one of the CE findings could be underestimated as a P1 lesion. In our study, small isolated angioectasia was revealed in 60.8% patients. Although the number of patients who used PEG with cold-water gut lavage was unknown, the number of cases with SB angioectasia could have been underestimated as P1 lesions due to either PEG with cold-water gut lavage, opiates, or both (7/46, 15.2%), in our study. Therefore, the possibility of a higher rebleeding risk in our study than in other studies with negative CE cannot be excluded. As mentioned above, rebleeding rate of a recent meta-analysis of negative CE is in accordance with our results. Therefore, we believe this effect may be insignificant. Third, repeat CE was not conducted for all patients with OGIB with rebleeding due to relatively high cost of CE. A policy providing reimbursement for CE and DBE costs to patients with OGIB has been implemented since 2015. The cost of CE and DBE, which was approximately $1000 and $1200–1500, respectively, before 2015, decreased to $200 and $500, respectively, after 2015. The cost of CT was maintained at approximately $200 during the study duration. In Korea, many patients with OGIB underwent CT because of its substantially lower cost than that of CE before 2015. However, since 2015, CT has been preferred due to convenience, rapid diagnosis, and availability. Therefore, we could not complete economic evaluation due to the considerable difference in CE and DBE costs during the study duration. Although 4 of 15 patients with rebleeding did not undergo additional CE evaluation, we tried performing SB evaluations using DBE, push enteroscopy, CTE, or angiography in most of the patients (73.3%). Fourth, we could not evaluate the role of DBE in our study as it was minimally used because of its higher cost; furthermore, in this study, we enrolled only patients with P0 and P1 lesions on CE. Patients with P0 lesions were treated conservatively without additional DBE. Because a high number of patients with P1 lesions (29/46, 63.0%) experienced OGIB before 2015, the use of the relatively expensive DBE was limited. Lastly, in 12 out of 84 patients, CE at the first bleeding episode was not examined completely. This could lead to missing lesions and influence the result of our study. However, in 10 of 11 patients (91%) with incomplete CE examination, CE reached the terminal ileum. It reached mid-ileum in only one patient due to gastric retention. Because the terminal ileum may be evaluated to some extent by ileocolonoscopy before CE, we believed that the effect of incomplete CEs was likely to be low. Despite limitations of our study, we tried to analyze the differences of clinical outcomes between P0 and P1 lesions in OGIB patients with negative CT and CE.

In conclusion, despite negative CT and CE results, patients with OGIB have a significant potential rebleeding risk. There was no significant difference of rebleeding rate between the P0 and P1 groups. Although the clinical significance of the P1 lesion was not evaluated differently from that of the P0 lesion, the P1 group, with CCI or low initial Hb, may require careful observation after the first bleeding episode.

## Figures and Tables

**Table 1:** Baseline characteristics of patients.

Variable	*N* = 84
Age, years	57.9 ± 19.9
Old age (>65 years)	30 (35.7)
Male gender	49 (58.3)
Type of first bleeding	
Overt	67 (79.8)
Occult	17 (20.2)
CCI	1.14 ± 1.68
DM	9 (10.7)
CVD	35 (41.7)
LC	5 (6.0)
ESRD	4 (4.8)
CVA	3 (3.6)
Medication at the first bleeding	
NSAIDs use	12 (14.3)
Aspirin use	10 (11.9)
Clopidogrel/cilostazol use	3 (3.6)/1 (1.2)
DOAC use	2 (2.4)
Warfarin use	2 (2.4)
Lowest Hb level at the first bleeding (g/dL)	8.6 ± 2.6
pRBC transfusion, pints	1.1 ± 1.9
Follow-up duration, months	35.9 ± 40.2

Variables are presented as mean ± SD or *n* (%). CCI, Charlson comorbidity index; DM, diabetes mellitus; CVD, cardiovascular disease; LC, liver cirrhosis; ESRD, end stage renal disease; CVA, cerebrovascular accident; NSAID, non-steroidal anti-inflammatory drug; DOAC, direct oral anticoagulant; Hb, hemoglobin; pRBC, packed red blood cell.

**Table 2:** Capsule endoscopy (CE)-related data.

Variable	*N* = 84
Type of CE	
PillCam (SB1)	8 (9.5)
PillCam (SB2)	31 (36.9)
PillCam (SB3)	34 (40.5)
MiroCam	11 (13.1)
Acceptable quality of images, *n* (%)	64 (76.2)
Arrival at cecum, *n* (%)	72 (85.7)
Transit time, mean, min	277.84 ± 208.31
Results of CE	
P1	46 (54.8)
Red spot(s) or small isolated angioectasia	22 (47.8)
Small isolated erosion	18 (39.1)
Red spot(s) or small isolated angioectasia + small isolated erosion	6 (13.0)
P0	38 (45.2)

**Table 3:** Comparison of characteristics between the P0 and P1 groups.

Variable	P0 (*N* = 38)	P1 (*N* = 46)	*p*-value
Age, years	56.8 ± 23.2	58.8 ± 17.1	0.665
Old age (>65 years)	14 (36.8)	16 (34.8)	>0.999
Male gender	20 (52.6)	29 (63.0)	0.379
Type of first bleeding			
Overt	29 (76.3)	38 (82.6)	0.588
Occult	9 (23.7)	8 (17.4)	
CCI	0.89 ± 1.33	1.34 ± 1.91	0.206
DM	6 (15.8)	3 (6.5)	0.288
CVD	16 (42.1)	19 (41.3)	>0.999
LC	2 (5.3)	3 (6.5)	>0.999
ESRD	0	4 (8.7)	0.123
CVA	1 (2.6)	2 (4.3)	>0.999
Medication at the first bleeding	9 (23.7)	14 (30.4)	0.624
NSAIDs use	5 (13.2)	7 (15.2)	>0.999
Aspirin use	1 (2.6)	9 (19.6)	0.020
Clopidogrel/cilostazol use	0	4 (8.7)	0.123
DOAC use	2 (5.3)	0	0.202
Warfarin use	1 (2.6)	1 (2.2)	>0.999
Lowest Hb level at the first bleeding	8.7 ± 3.0	8.5 ± 2.3	0.694
pRBC transfusion, pints	0.9 ± 1.5	1.3 ± 2.3	0.413
Rebleeding	7 (18.4)	8 (17.4)	>0.999
Time interval between the first episode and rebleeding	11.4 ± 7.6	42.3 ± 42.0	0.078
Follow-up duration, months	36.2 ± 39.8	35.6 ± 41.1	0.942

Variables are presented as mean ± SD or *n* (%). CCI, Charlson comorbidity index; DM, diabetes mellitus; CVD, cardiovascular disease; LC, liver cirrhosis; ESRD, end stage renal disease; CVA, cerebrovascular accident; NSAID, non-steroidal anti-inflammatory drug; DOAC, direct oral anticoagulant; Hb, hemoglobin; pRBC, packed red blood cell.

**Table 4:** Univariate and multivariate analyses of factors associated with rebleeding in OGIB patients with negative CT and CE.

	Univariate Analysis		Multivariate Analysis	
	HR (95% CI)	*p*-Value	HR (95% CI)	*p*-Value
P0 group				
Old age >65 years	11.244 (1.349–93.699)	0.025		
Aspirin use	31.937 (1.995–511.293)	0.014		
Initial Hb <8 g/dL	6.707 (0.800–56.216)	0.079		
Reuse of bleeding-related drugs	6.655 (1.078–41.091)	0.041		
P1 group				
CCI	1.775 (1.239–2.542)	0.002	2.019 (1.158–3.519)	0.013
LC	6.058 (1.095–33.525)	0.039		
Initial Hb <8 g/dL	12.121 (1.483–99.082)	0.020	15.085 (1.182–192.514)	0.037
pRBC transfusion (>2 pints)	6.215 (0.992–38.937)	0.051		

OGIB, obscure gastrointestinal bleeding; CT, computed tomography CE, capsule endoscopy; HR, hazard ratio; Hb, hemoglobin; CCI, Charlson comorbidity index; LC, liver cirrhosis pRBC, packed red blood cell.

## Data Availability

The data presented in this study are available on request from the corresponding author. The data are not publicly available due to privacy.

## Supplementary Materials

The following are available online at https://www.mdpi.com/article/10.3390/diagnostics11040657/s1, Table S1. Comparison of characteristics between the non-rebleeding and rebleeding groups; Table S2. Univariate and multivariate analyses of factors associated with rebleeding in OGIB patients with negative CT and CE
