# Supplementary material for: Clinical Outcomes between P1 and P0 Lesions for Obscure Gastrointestinal Bleeding with Negative Computed Tomography and Capsule Endoscopy
Source: Diagnostics (Basel). 2021 Apr 6;11(4):657. doi: 10.3390/diagnostics11040657 (PMC8067371; doi:10.3390/diagnostics11040657)
Supplement: Supplementary file 1 [file diagnostics-11-00657-s001.pdf]

diagnostics

MDPI

Article

Clinical Outcomes between P1 and P0 lesions for Obscure Gastrointestinal Bleeding with Negative Computed Tomography and Capsule Endoscopy

Young Kyu Cho [1,†], Heesu Park [1,†], Jung Rock Moon [1], Seong Ran Jeon [1,*], Hyun Gun Kim [1], Tae Hee Lee [1], Jun-seok Park [1], Jin-Oh Kim [1], Joon Seong Lee [1], Hyeon Jeong Goong [1], Bong Min Ko [1] and Suyeon Park [2]

[1] Digestive Disease Center, Institute for Digestive Research,Soonchunhyang University College of Medicine, 59 Daesagwan-ro, Yongsan-Gu, Seoul 04401, Korea; 109133@schmc.ac.kr (Y.K.C.); phs759@hanmail.net (H.P.); ukino816@gmail.com (J.R.M.); medgun@schmc.ac.kr (H.G.K.); iman0825@naver.com (T.H.L.); junspark@schmc.ac.kr (J.P.); jokim@schmc.ac.kr (J.-O.K.); joonlee@schmc.ac.kr (J.S.L.); goong@schmc.ac.kr (H.J.G.); kopa9445@schmc.ac.kr (B.M.K.)

[2] Department of biostatistics, Soonchunhyang University College of Medicine, 59 Daesagwan-ro, Yongsan-Gu, Seoul 04401, Korea; suyeon1002@schmc.ac.kr

* Correspondence: 94jsr@hanmail.net; Tel.: +82-2-709-9202; Fax: +82-2-709-9581

† Both authors contributed equally to this manuscript

Citation: Cho, Y.K.; Park, H.; Moon, J.R.; Jeon, S.R.; Kim, H.G.; Lee, T.H.; Park, J.; Kim, J.-O.; Lee, J.S.; Goong, H.J.; et al. Clinical Outcomes between P1 and P0 lesions for Obscure Gastrointestinal Bleeding with Negative Computed Tomography and Capsule Endoscopy. *Diagnostics* 2021, *11*, x. https://doi.org/10.3390/xxxxx

Academic Editor: Hajime Isomoto

Received: 24 February 2021

Accepted: 1 April 2021

Published: date

Publisher's Note: MDPI stays neutral with regard to jurisdictional claims in published maps and institutional affiliations.

Copyright: © 2021 by the authors. Licensee MDPI, Basel, Switzerland. This article is an open access article distributed under the terms and conditions of the Creative Commons Attribution (CC BY) license (http://creativecommons.org/licenses/by/4.0/).

Table S1. Comparison of characteristics between the non-rebleeding and rebleeding groups.

Variable	Non-rebleeding (*N* = 69)	Rebleeding (*N* = 15)	*p*-value
Age, years	56.5 ± 19.3	64.0 ± 22.7	0.192
Old age (>65 y)	21 (30.4)	9 (60.0)	0.040
Male gender	40 (58.0)	9 (60.0)	>0.999
Type of first bleeding, *n* (%)			
Overt	55 (79.7)	12 (80.0)	>0.999
Occult	14 (20.3)	3 (20.0)	
CCI	0.96 ± 1.49	2.00 ± 2.23	0.028
DM	5 (7.2)	4 (26.7)	0.049
CVD	27 (39.1)	8 (53.3)	0.390
LC	3 (4.3)	2 (13.3)	0.216
ESRD	3 (4.3)	1 (6.7)	0.552
CVA	2 (2.9)	1 (6.7)	0.450
Medication at the first bleeding	19 (27.5)	4 (26.7)	>0.999
NSAIDs use	12 (17.4)	0	0.113
Aspirin use	7 (10.1)	3 (20.0)	0.374
Clopidogrel/cilostazol use	2 (2.9)	2 (13.3)	0.145
DOAC use	1 (1.4)	1 (6.7)	0.327
Warfarin use	2 (2.9)	0	>0.999
Lowest Hb level at the first bleeding	9.0 ± 2.0	6.7 ± 1.7	0.001
pRBC transfusion, pints	0.9 ± 2.0	2.2 ± 1.3	0.021
Reuse of bleeding-related drugs	10 (14.5)	4 (26.7)	0.264
NSAIDs use	1 (1.4)	0	>0.999
Aspirin use	5 (7.2)	3 (20.0)	0.580
Clopidogrel/cilostazol use	3 (4.3)	0	0.505
DOAC use	0	1 (6.7)	0.286
Warfarin use	3 (4.3)	0	0.505
Follow-up duration, months	34.4 ± 39.6	27.9 ± 34.0	0.557

Variables are presented as mean ± SD or *n* (%). CCI, Charlson comorbidity index; DM, diabetes mellitus; CVD, cardiovascular disease; LC, liver cirrhosis; ESRD, end stage renal disease; CVA, cerebrovascular accident; NSAID, non-steroidal anti-inflammatory drug; DOAC, direct oral anti-coagulant; Hb, hemoglobin; pRBC, packed red blood cell.

Table S2. Univariate and multivariate analyses of factors associated with rebleeding in OGIB patients with negative CT and CE.

	Univariate analysis		Multivariate analysis	
	**HR (95% CI)**	***p*-value**	**HR (95% CI)**	***p*-value**
Old age (>65 y)	3.272 (1.153–9.288)	0.026		
Male gender	1.015 (0.361–2.856)	0.978		
CCI	1.394 (1.078–1.803)	0.011	1.366 (1.016–1.836)	0.039
DM	2.758 (0.875–8.688)	0.083		
CVD	0.466 (0.167–1.302)	0.145		
LC	2.188 (0.481–9.952)	0.311		
ESRD	1.711 (0.221–13.236)	0.607		
CVA	2.735 (0.352–21.276)	0.336		
NSAIDs use	0.041 (0.000–53.814)	0.383		
Aspirin use	1.902 (0.535–6.761)	0.321		
Anti-PLT use	1.904 (0.422–8.580)	0.402		
Warfarin use	0.047 (0.000–16939.544)	0.639		
DOAC use	5.297 (0.650–43.160)	0.119		
Overt OGIB	0.967 (0.272–3.482)	0.967		
Initial Hb <8 g/dL	11.750 (2.589–53.326)	0.001	9.001 (1.875–43.202)	0.006
Transfusion (>2 pints)	3.488 (1.148–10.601)	0.028		
Arrival at cecum	0.770 (0.164–3.610)	0.741		
Acceptable image	0.197 (0.026–1.497)	0.116		
P1 finding	1.021 (0.369–2.821)	0.969		
Reuse of bleeding-related drugs	1.998 (0.634–6.295)	0.237		

CE, capsule endoscopy; CT, computed tomography; HR, hazard ratio; CCI, Charlson comorbidity index; DM, diabetes mellitus; CVD, cardiovascular disease; LC, liver cirrhosis; ESRD, end stage renal disease; CVA, cerebrovascular accident; NSAID, non-steroidal anti-inflammatory drug; PLT, platelet; DOAC, direct oral anticoagulant; OGIB, obscure gastrointestinal bleeding; Hb, hemoglobin.
